# Pathophysiology and causes of hyperkalemia: unraveling causes beyond kidney dysfunction

**DOI:** 10.1007/s10157-025-02711-x

**Published:** 2025-06-11

**Authors:** Takuya Fujimaru, Kazuhito Hirose, Masahiko Yazawa, Masahiko Nagahama, Csaba P. Kovesdy

**Affiliations:** 1 Department of Nephrology, St. Luke’s International Hospital, 9-1 Akashi-Cho, Chuo-Ku, Tokyo, 104-8560 Japan; 2 Department of General Medicine, Tsukuba Medical Center Hospital, Tsukuba, Japan; 3 Department of Internal Medicine, Yokohama General Hospital, Yokohama, Japan; 4 Division of Nephrology and Hypertension, Department of Internal Medicine, St. Marianna University School of Medicine, Kawasaki, Japan; 5 Division of Nephrology, Department of Medicine, University of Tennessee Health Science Center, Memphis, TN USA; 6 Memphis Veterans Affairs Medical Center, Nephrology Section, Memphis, TN USA

**Keywords:** Hyperkalemia, Potassium, Dyskalemia, Pseudohyperkalemia, Mortality

## Abstract

This review article series on water and electrolyte disorders is based on the “Electrolyte Winter Seminar” that is held annually for young nephrologists in Japan. This seminar includes lively discussions based on cases, which have been partly included in this series as self-assessment questions. In this fourth article of the series, we have focused on the pathophysiology of potassium and the diagnosis of hyperkalemia. Hyperkalemia is associated with increased overall mortality, cardiovascular mortality, hospitalization, and progression to end-stage renal disease. Although most patients with hyperkalemia exhibit impaired kidney function, some exhibit normal kidney function. Therefore, accurately diagnosing the underlying cause of hyperkalemia is crucial for its appropriate management. In this review, we have first discussed the pathophysiology of potassium regulation. We have then highlighted the causes of hyperkalemia other than chronic kidney disease, including pseudohyperkalemia, which is often overlooked in clinical practice, and hypoaldosteronism, which can cause hyperkalemia even in patients with normal or mildly impaired kidney function. Finally, we have shared practical “Tips and Pearls” on hyperkalemia for clinicians that are applicable in daily practice.

## Introduction

Hyperkalemia is associated with an increased mortality rate [1]. Although most patients with hyperkalemia exhibit impaired kidney function, some exhibit normal kidney function. Therefore, it is crucial to accurately diagnose the underlying cause of hyperkalemia. Inappropriate potassium restriction and potassium-binding medications may be prescribed, especially in patients with pseudohyperkalemia, potentially leading to adverse events such as hypokalemia, constipation, and unnecessary medical costs. In addition, improper management during fasting conditions in patients on dialysis can lead to life-threatening hyperkalemia. This review outlines the pathophysiology of potassium and the causes of hyperkalemia, primarily those other than kidney dysfunction.

### Definition and prevalence of hyperkalemia

Hyperkalemia is defined as a plasma potassium concentration of ≥ 5.0 mEq/L [2, 3]. The kidneys play a crucial role in potassium homeostasis. Consequently, as kidney function declines, the prevalence of hyperkalemia increases [4–7]. A report based on Japanese claims data found that the prevalence of hyperkalemia (> 5.1 mEq/L) increased with worsening chronic kidney disease (CKD) stage: 7.2% in stage 1, 10.3% in stage 2, 13.2% in stage 3a, 24.6% in stage 3b, 43.7% in stage 4, and 51.2% in stage 5 [5]. Although the prevalence of hyperkalemia varies widely depending on comorbidities, particularly diabetes, heart failure, CKD, and concomitant medications, it follows a linear trend with the decline in renal function. Therefore, hyperkalemia management is crucial in patients with CKD.

### Consequences of hyperkalemia

The presence of hyperkalemia is associated with an increased risk of mortality. A large-scale observational study using Japanese claims data demonstrated a linear relationship between increasing serum potassium levels and higher mortality risk at 3 years across CKD stages G3a, G3b, G4, and G5 [5]. In this study, using a serum potassium range of 3.6–5.0 mEq/L as the reference, potassium levels of 5.1–5.4 mEq/L, which are typically considered manageable in CKD care, were associated with a significantly increased risk of mortality (hazard ratio [HR]: 7.6). Furthermore, potassium levels between 5.5 and 5.9 mEq/L were associated with an even higher mortality risk (HR: 10.6). Moreover, this study demonstrated a U-shaped relationship between potassium abnormalities and 1-year mortality risk. This trend has been confirmed by a meta-analysis of global cohort studies. Compared with a reference level of 4.2 mEq/L, the overall adjusted HR for all-cause mortality was 1.22 (95% confidence interval [CI] 1.15–1.29) at a serum potassium level of 5.5 mEq/L and 1.49 (95% CI 1.26–1.76) at a serum potassium level of 3.0 mEq/L [7]. This meta-analysis also showed that hyperkalemia was associated with cardiovascular mortality and progression to end-stage kidney disease (ESKD). The association between hyperkalemia and mortality could be explained by the induction of malignant arrhythmias and their consequences, such as hypotension, myocardial ischemia, and sudden cardiac death [7]. While there is no randomized control trial to conclusively prove that hyperkalemia is a cause of death or other major adverse cardiovascular events, the robust observational evidence and the plausible pathophysiology are sufficient for clinicians and guidelines to consider hyperkalemia an important risk factor. Moreover, although there is currently no clear consensus on the optimal target range for serum potassium levels, the Japanese CKD Clinical Practice Guidelines 2018 and 2023 recommend maintaining serum potassium levels between 4.0 and < 5.5 mEq/L in patients with CKD, as this may reduce the risk of all-cause mortality and cardiovascular events [8].

Hyperkalemia is associated with an increased number of hospitalizations (1.2 to 1.6 times greater) compared with normokalemia [9]. In addition, the total medical costs for patients with hyperkalemia are significantly higher than those for patients with normokalemia. A Japanese cohort study found that the frequency of repeat hyperkalemic episodes, rather than the severity of hyperkalemia, was associated with higher long-term healthcare costs [10].

## Pathophysiology of hyperkalemia

### Potassium distribution

The total amount of potassium in the human body is estimated to be approximately 3000–3500 mmol (50–75 mEq/kg) [11]. Unlike sodium, which is present in large amounts in the extracellular fluid (ECF), approximately 98% of the total body potassium is located in the intracellular fluid (ICF), primarily in the muscle. Serum potassium levels are tightly regulated through intracellular–extracellular shifts and urinary excretion. According to the Ministry of Health, Labour and Welfare’s Dietary Intake Standards for Japanese (2020 Edition), the recommended daily potassium intake for Japanese adults is 2000–2500 mg (51.2–63.9 mmol). In healthy adults, approximately 90%–95% of the ingested potassium is excreted in the urine, while around 5–10% is excreted in the stool. However, fecal potassium excretion is about three times higher in patients on hemodialysis than in healthy individuals, reaching up to 35%–80% of the dietary potassium intake (up to 3000 mg per day) in some patients on hemodialysis [12, 13]. Consistent with this, a case report described a hemodialysis patient who developed severe hyperkalemia during management with a temporary ileostomy following ischemic colitis. After restoration of bowel continuity, serum potassium levels returned to baseline, accompanied by evidence of increased fecal potassium excretion, suggesting that impaired colonic potassium secretion contributed to the hyperkalemia [14].

### Factors influencing the intracellular and extracellular distribution of potassium

The major physiological factors that stimulate the movement of potassium from the ECF compartment to the ICF compartment are insulin and epinephrine. The regulation of extrarenal potassium handling by insulin and β₂-adrenergic agonists is primarily mediated through the stimulation of Na⁺/K⁺-ATPase activity in skeletal muscle cells. In contrast to β-adrenergic stimulation, α-adrenergic stimulation promotes the movement of potassium from the ICF compartment to the ECF compartment, leading to an increase in plasma potassium levels [15]. However, the cellular mechanisms underlying this process remain unknown.

**Fig. 1:**
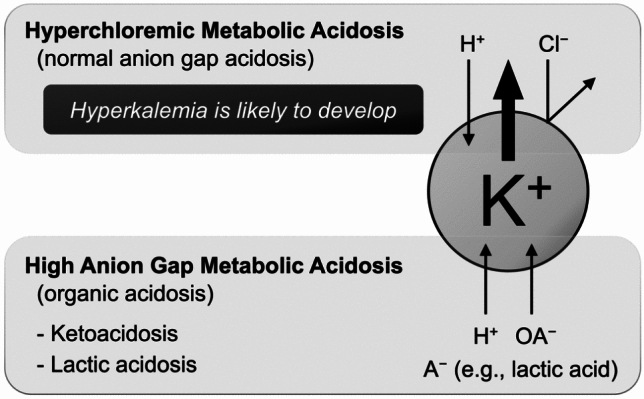
Mechanism of hyperkalemia in metabolic acidosis: normal anion gap vs. high anion gap. Organic acid ions (OA^−^) are cell-permeable, whereas chloride ions (Cl^–^) are not. K^+^, potassium ion; H^+^, proton

Acid–base disturbances also affect the potassium distribution in the body. Metabolic acidosis, in particular, promotes potassium efflux from the cells. However, the impact of metabolic acidosis on plasma potassium levels depends on the underlying cause: hyperchloremic metabolic acidosis (normal anion gap acidosis) or high anion gap acidosis (organic acidosis) (Fig. 1). The effect of acidosis on potassium homeostasis depends on whether the proton enters the cells with or without accompanying anions. If a proton enters the cells without an accompanying anion, potassium moves out into the extracellular space. In contrast, when a proton enters along with an anion, potassium remains within the cells, preventing an extracellular shift of potassium. Cells have low permeability to chloride; therefore, in hyperchloremic metabolic acidosis—including renal tubular acidosis and diarrhea-related acidosis—anions like chloride cannot enter the cells, leading to potassium efflux into the extracellular space. In contrast, the cells are highly permeable to organic anions. As a result, in conditions involving organic acids, such as ketoacidosis or lactic acidosis, the proton is transported into the cells along with its conjugate anions, minimizing potassium movement [16]. Therefore, only hyperchloremic metabolic acidosis significantly impacts serum potassium levels, whereas high anion gap acidosis does not cause notable potassium shifts.

### Mechanism of potassium excretion in the urinary tract

**Fig. 2:**
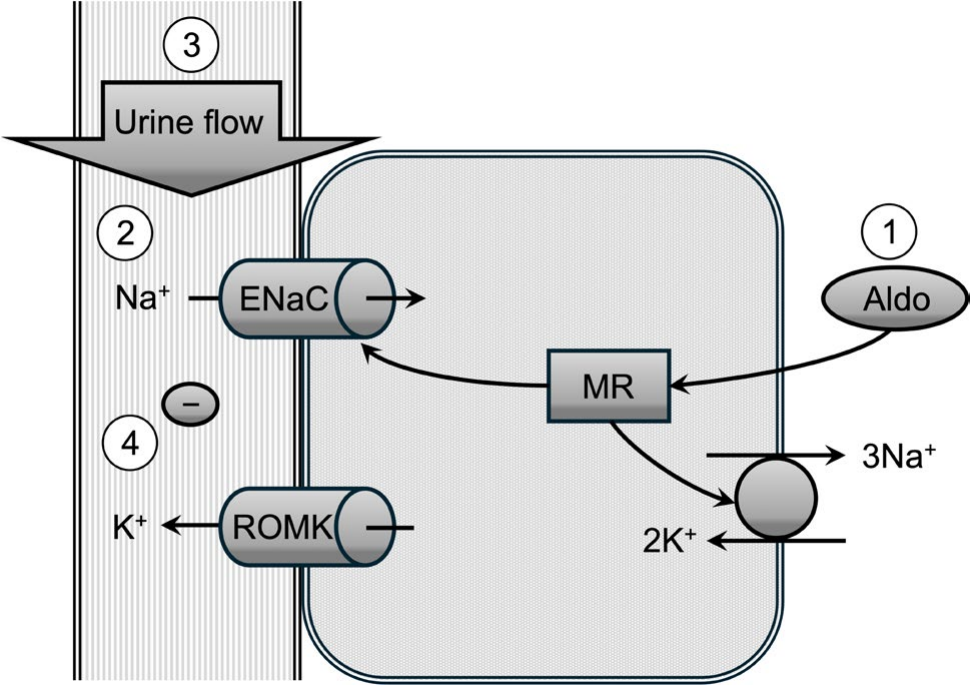
Mechanisms of potassium excretion in the principal cells of the renal cortical collecting duct. The circled numbers represent the four factors that promote renal potassium excretion: (1) aldosterone action, (2) sodium flow, (3) urine flow, and (4) luminal negative charge. Aldo, aldosterone; ENaC, epithelial sodium channels; MR, mineralocorticoid receptor; ROMK, renal outer medullary potassium channel

Most of the ingested potassium is excreted via the kidneys, with the site of excretion localized to the aldosterone-sensitive distal nephron. In particular, the principal cells of the cortical collecting duct play a major role in potassium secretion. Four main factors—aldosterone action, sodium flow, urine flow, and luminal negative charge—promote potassium excretion in the distal nephron (Fig. 2). Aldosterone stimulates epithelial sodium channel (ENaC) activity via mineralocorticoid receptors, increasing both the number of channels and their probability of opening [17].

Furthermore, aldosterone enhances Na⁺/K⁺-ATPase activity in collecting duct cells, and its downstream kinase, serum- and glucocorticoid-regulated kinase 1 (SGK1), increases the surface expression of the renal outer medullary potassium channel (ROMK; also known as Kir1.1) by phosphorylating serine 44 [18], thereby stimulating potassium secretion into the tubular lumen. Sodium entering the collecting duct is reabsorbed through ENaC, creating an electrically negative luminal environment, which facilitates potassium secretion via the renal outer medullary potassium channel. Thus, potassium excretion is influenced not only by the amount of sodium delivered to the renal tubular lumen but also by an increase in the luminal negative charge, such as that caused by metabolic alkalosis or alkaline agent administration. In addition, increased urine flow enhances potassium excretion by both increasing sodium delivery to the distal nephron and amplifying the potassium concentration gradient between the tubular lumen and principal cells.

### Response to oral potassium loading

Because potassium intake promotes urinary potassium excretion, hyperkalemia due to excessive potassium intake rarely occurs in healthy individuals. In a study where potassium intake in healthy adults was increased from 100 mEq/day to 400 mEq/day, urinary potassium excretion reached 300 mEq/day by the second day of increased intake, and the serum potassium concentration increased only to 4.8 mEq/L [19]. This indicates that an increase in blood potassium concentration stimulates aldosterone secretion and enhances potassium excretion from the principal cells of the collecting duct in an aldosterone-independent manner [20]. Interestingly, potassium loading itself has been reported to dephosphorylate the sodium–chloride cotransporter (NCC) in the distal tubule, increasing sodium delivery to the collecting duct and thereby promoting potassium excretion in the principal cells [20]. This phenomenon, referred to as the “gut factor,” represents a gastrointestinal–renal potassium signaling axis and mediates potassium excretion independently of changes in serum potassium concentration and aldosterone [21, 22].

## Cause of hyperkalemia

Since the kidneys are the primary organs responsible for potassium excretion, the causes of hyperkalemia can be more clearly understood by categorizing them into kidney dysfunction and other conditions (Table 1).

**Table 1:** Mechanisms and causes of hyperkalemia

Pseudohyperkalemia	
Localized potassium release at the blood sampling site	
Hemolysis, fist clenching	
Sample contamination with K^+^ EDTA	
Potassium efflux inside the blood collection tube	
Thrombocytosis, leukocytosis, familial pseudohyperkalemia (*ABCB6*, *SLC4 A1*, *PIEZO1*)	

Other than kidney dysfunction	
Abnormal potassium distribution	
Insulin deficiency, beta-blockers, hyperchloremic metabolic acidosis, hyperkalemic periodic paralysis, hyperosmolar substances in ESKD/AKI, fasting hyperkalemia in ESKD	
Decreased kidney excretion due to hypoaldosteronism: Type IV RTA	
Diseases: diabetic nephropathy, PHAII/Gordon syndrome, adrenal insufficiency	
Medications: ACEi, ARB, direct renin inhibitors, aldosterone synthase inhibitors, NSAIDs, heparin, MRA, trimethoprim, pentamidine, CNI	
Abnormal potassium release from cells	
Rhabdomyolysis, tumor lysis syndrome	

Kidney dysfunction	
CKD, AKI	

*ACEi* Angiotensin-converting enzyme inhibitor, *AKI* Acute kidney injury, *ARB* Angiotensin II receptor blockers, *CKD* Chronic kidney disease, *CNI* Calcineurin inhibitors, *ESKD* End-stage kidney disease, *MRA* Mineralocorticoid receptor antagonist, *NSAIDs* Nonsteroidal anti-inflammatory drugs, *PHAII* Pseudohypoaldosteronism type 2, *RTA* Renal tubular acidosis

### Causes of hyperkalemia other than kidney dysfunction

#### Pseudohyperkalemia

Pseudohyperkalemia refers to conditions in which laboratory potassium measurements are falsely elevated, despite normal physiological potassium levels in the bloodstream. These discrepancies can lead to unnecessary treatment, emphasizing the importance of careful interpretation of the potassium test results. Clinicians should be aware of these artifacts. Pseudohyperkalemia should be suspected when an elevated serum potassium level is observed in the absence of identifiable risk factors, such as excessive potassium intake, kidney dysfunction, diabetes, or medication-induced hyperkalemia. Notably, the affected patients exhibited no electrocardiogram abnormalities or muscular symptoms because the elevated potassium levels were confined to the test tube and did not reflect the patient’s actual state. Some causes of pseudohyperkalemia are described below.

##### Localized potassium release at the blood sampling site

Extracellular potassium levels are largely influenced by hematologic cells, as 98% of the body’s potassium resides intracellularly [23]. Hemolysis during blood collection, which is caused by fine-gauge needles, excessive suction, vigorous shaking, or improper centrifugation, commonly leads to pseudohyperkalemia. A concurrent elevation in lactate dehydrogenase (LDH), aspartate aminotransferase, and serum iron levels can help distinguish hemolysis-related pseudohyperkalemia from true hyperkalemia.

Fist clenching during tourniquet use is another major cause of pseudohyperkalemia. Repeated handgrip exercises while a tourniquet is applied can increase serum potassium levels by up to 1.4 mEq/L [24]. Although the tourniquet itself has minimal effect, it impairs the washout of potassium released from exercising muscle [25]. Therefore, patients should be instructed to avoid fist clenching or repeated handgrip exercises during blood collection.

##### Ethylenediaminetetraacetic acid (EDTA) contamination

EDTA contamination from improper handling of anticoagulant tubes can falsely elevate potassium levels [26]. Possible contamination scenarios include blood collection from an EDTA-2 K tube or contamination of the syringe tip when injecting blood into an EDTA-2 K tube. When such contamination is significant, EDTA chelates divalent and trivalent ions, leading to decreased levels of zinc, magnesium, and calcium. Because zinc serves as a coenzyme for alkaline phosphatase (ALP), a reduction in ALP levels may also occur [26]. However, in cases of mild contamination, differentiation is only possible by directly measuring EDTA-2 K. In such cases, retesting is recommended.

##### Leukocytosis and thrombocytosis

Patients with myeloproliferative disorders may exhibit pseudohyperkalemia due to potassium release from white blood cells or platelets during coagulation [27]. Differentiation can be achieved by collecting blood in heparinized tubes and measuring potassium in the plasma. Alternatively, immediate transport of the blood sample to the laboratory can help prevent artifactual potassium elevation and ensure accurate measurement.

##### Potassium leakage from red blood cells in the cold and long-term storage in normal subjects

Delays in sample processing, particularly at low temperatures (4 °C), can lead to potassium leakage from the red blood cells, resulting in spurious hyperkalemia. This issue is especially relevant when blood samples collected in outpatient clinics are transported to a remote central laboratory for analysis, especially during cold seasons. Consequently, a phenomenon known as “seasonal pseudohyperkalemia” may occur [28].

**Fig. 3:**
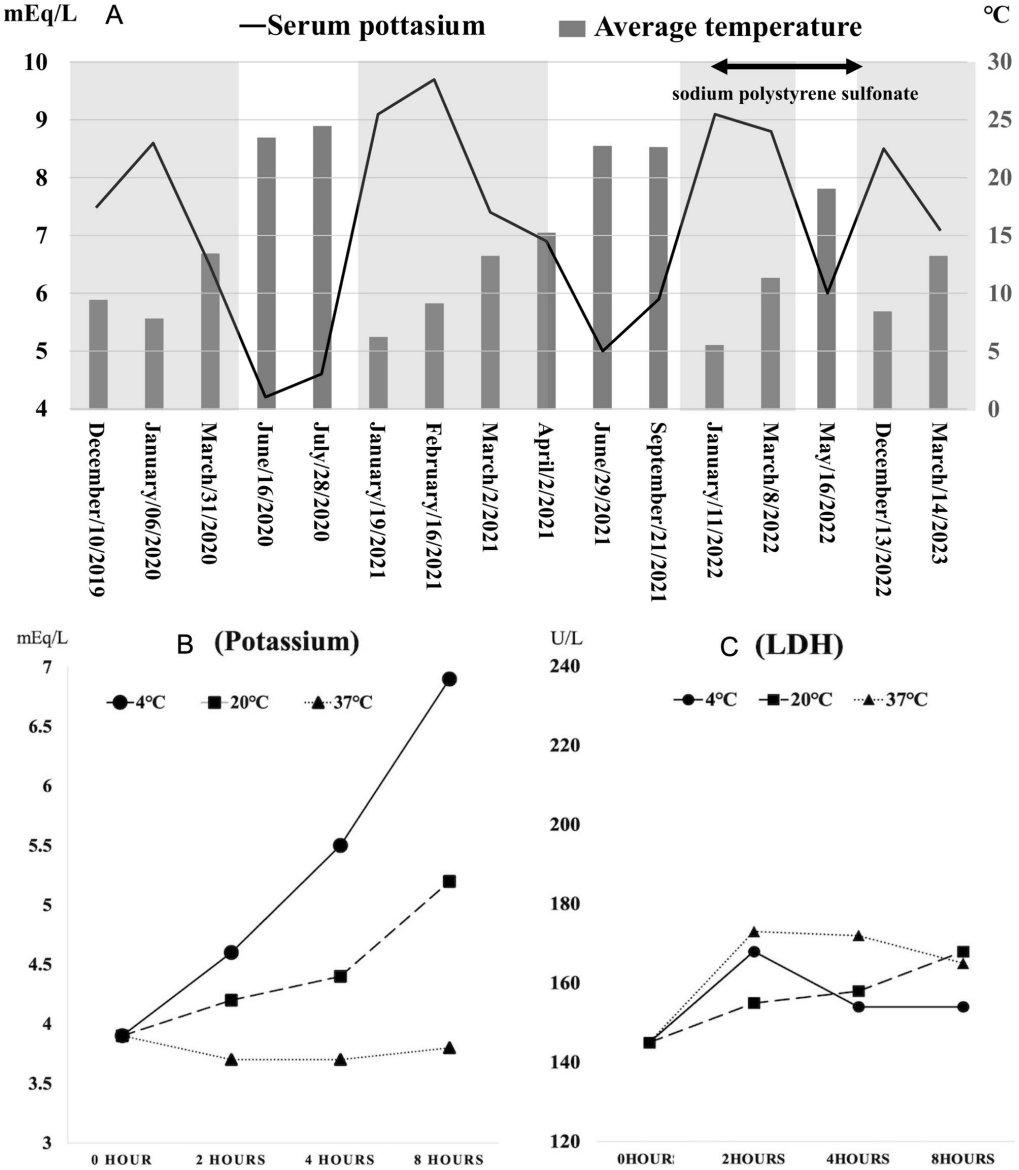
Clinical and laboratory features suggesting familial pseudohyperkalemia. **A** Serum potassium trajectory based on the examination date and average monthly temperatures in Yokohama, Japan. The gray-shaded area indicates the cold season (October to April). **B** Serum potassium levels measured under different storage temperatures (4 °C, 20 °C, and 37 °C) and durations. **C** Serum lactate dehydrogenase (LDH) levels under the same storage conditions. Modified from CEN Case Reports 2024[29]

**Self-assessment question 1** A woman in her 70 s with type 2 diabetes and dyslipidemia presented with refractory hyperkalemia on routine blood tests at the clinic over several years. She was taking only an anagliptin–metformin combination tablet and rosuvastatin. As shown in Fig. 3A, her potassium levels fluctuated between 4.5 and 9.7 mEq/L. Despite these elevated potassium levels, she remained asymptomatic, with stable blood pressure and no signs of fatigue or muscle weakness. Her electrocardiogram was also normal. At a tertiary hospital, her serum potassium level was measured at 3.7 mEq/L, her complete blood count was unremarkable, and her estimated glomerular filtration ratio (eGFR) was 89.3 mL/min/1.73 m^2^. The other electrolytes—including calcium and magnesium—were within normal ranges. Specific examinations revealed no evidence of hypoaldosteronism or metabolic acidosis. To further investigate her potassium abnormalities, nephrologists measured her serum potassium and LDH levels under various storage conditions, including different temperatures and storage durations, as shown in Fig. 3B and C.

**Question: What is the most likely cause of this patient’s hyperkalemia?**

A. Adrenal insufficiency.

B. Familial pseudohyperkalemia.

C. Hemolysis-induced hyperkalemia.

D. Laboratory contamination with EDTA.

The correct answer is B.

##### Familial pseudohyperkalemia (FP)

Figure 3A demonstrates fluctuating serum potassium levels at the clinic, while Fig. 3B and C show a temperature-dependent increase in potassium without a corresponding increase in LDH, strongly suggesting FP [29]. FP is a rare, autosomal dominant disorder caused by mutations in *ABCB6* and *SLC4 A1*, leading to abnormal potassium leakage from red blood cells at lower temperatures and even at room temperature (e.g., 20 °C). The storage conditions of the blood samples at the clinic, including low temperature and prolonged storage, induced pseudohyperkalemia in these patients. In contrast, at the tertiary hospital, the interval from blood collection to examination was within a few minutes to 1 h, preventing potassium leakage from red blood cells even in patients with FP, allowing for proper differentiation. Unlike EDTA contamination (D), FP does not cause calcium or magnesium depletion. The patient’s normal LDH levels do not support hemolysis-induced hyperkalemia (C). Stable blood pressure and absence of fatigue did not support adrenal insufficiency (A). This case is based on a *CEN Case Report* published in 2024 [29].

In FP, misdiagnosis can result in repeated and unnecessary investigations. FP also poses a transfusion risk, as affected donors may exhibit elevated potassium levels in stored blood, particularly at low temperatures [30, 31]. This is especially hazardous for neonates and critically ill patients. Therefore, proper recognition of FP is crucial to avoid mismanagement and ensure patient safety.

#### Hyperkalemia other than kidney dysfunction, except pseudohyperkalemia

##### Abnormal potassium distribution

As mentioned above, insulin and sympathetic stimulation facilitate the movement of potassium from the extracellular to the intracellular space. Consequently, insulin deficiency and β-blocker administration can lead to hyperkalemia. In addition, hyperchloremic metabolic acidosis—in which only protons enter cells while potassium is released—also contributes to hyperkalemia.

Hyperkalemic periodic paralysis is a congenital disorder characterized by intracellular and extracellular potassium distribution abnormalities. It is an autosomal dominant condition caused by a gain-of-function missense variant in the *SCN4 A* gene, which encodes a sodium channel in skeletal muscle cells [32]. In patients with hyperkalemic periodic paralysis, episodes of weakness and paralysis are typically triggered by exposure to cold, rest following pain relief, fasting, or the ingestion of small amounts of potassium.

Hyperkalemia can also be induced by the administration of hyperosmolar substances, such as immunoglobulins [33] and radiocontrast agents [34]. The osmotic loading of these substances causes the osmotic movement of water out of the cell. Consequently, the resulting increase in the intracellular potassium concentration due to water loss promotes passive potassium efflux through the plasma membrane’s potassium channels. In addition, potassium is thought to move through the water pores in the cell membrane via the frictional forces between the solvent (water) and the solute. However, hyperkalemia is unlikely to occur if renal potassium excretion is normal, as most reported cases involve patients with kidney failure.

**Self-assessment question 2** A 72-year-old anuric maintenance hemodialysis patient with a 5-year history of dialysis underwent ablation for atrial fibrillation. After the three-hour-long procedure, the patient was unable to eat. Following the ablation, the patient’s heart rate and blood pressure dropped to 30 bpm and 74/40 mmHg (normal: 140/80 mmHg), respectively. A blood test revealed a serum potassium level of 6.6 mEq/L. What is the most likely cause of this patient’s hyperkalemia?

**Question:**

A. Postoperative nonsteroidal anti-inflammatory drug (NSAID) administration.

B. Consumption of a large amount of raw vegetables and fresh fruit for dinner the previous night.

C. Fasting hyperkalemia.

D. Insufficient dialysis due to hemodialysis not being performed the previous day.

The correct answer is C.

##### Fasting hyperkalemia

Although not widely recognized, dialysis patients can develop hyperkalemia after prolonged fasting (≥ 8 h), a condition known as “fasting hyperkalemia” [35–37]. Fasting hyperkalemia occurs due to the suppression of endogenous insulin secretion during fasting, leading to the shift of potassium from the ICF to the ECF. Unlike healthy individuals who can readily excrete excess extracellular potassium in the urine, dialysis patients lack this adaptive mechanism and are therefore prone to hyperkalemia. Fasting hyperkalemia may be observed when dialysis patients fast in preparation for surgery or diagnostic studies or when they are unable to eat. Fasting hyperkalemia in patients with ESKD can be prevented through intravenous glucose administration, which stimulates insulin secretion. Furthermore, in diabetic dialysis patients with impaired endogenous insulin secretion, insulin must be coadministered with glucose.

##### Decreased potassium excretion from the kidneys due to hypoaldosteronism

Even in the absence of kidney dysfunction, hyperkalemia can occur due to a reduction in aldosterone activity, which promotes potassium excretion from the principal cells of the collecting ducts (Fig. 2). The widely used term “hypoaldosteronism” encompasses both reduced aldosterone secretion and decreased responsiveness to aldosterone. In addition to hyperkalemia, hypoaldosteronism is associated with hyperchloremic metabolic acidosis, known as type 4 renal tubular acidosis. This metabolic acidosis results from decreased urinary ammonia excretion. Therefore, measuring the urine anion and osmolality gaps enables the estimation of urinary ammonium excretion, which has diagnostic utility [38, 39].

In patients with diabetes, decreased plasma renin activity leads to hypoaldosteronism. The underlying cause of hyporeninemia is thought to be either a defect in the conversion of the precursor prorenin to its active form renin [40] or the suppression of renin secretion due to diabetes-associated fluid overload.

Various drugs are known to cause hyperkalemia due to hypoaldosteronism. Drugs that directly or indirectly inhibit aldosterone production—such as angiotensin-converting enzyme inhibitor (ACEi), angiotensin II receptor blocker (ARB), direct renin inhibitors, and aldosterone synthase inhibitors [41, 42]—cause hypoaldosteronism and induce hyperkalemia [43]. Prostaglandins stimulate renin production. Therefore, NSAIDs, which are prostaglandin inhibitors, indirectly reduce aldosterone production by inhibiting renin production. Heparin has been shown to directly inhibit aldosterone production in the renal cortex by reducing the number and affinity of angiotensin II receptors on glomerular zone cells [44].

Some drugs induce hypoaldosteronism by reducing the responsiveness to aldosterone. mineralcorticoid receptor antagonist (MRA) competes with aldosterone for receptor sites. Two antibiotics, trimethoprim (usually given as trimethoprim sulfamethoxazole) and pentamidine, can also induce hyperkalemia by closing ENaC in the collecting ducts [45].

Pseudohypoaldosteronism type 2 (PHAII), also known as Gordon’s syndrome, is characterized by hypertension, hyperkalemia, metabolic acidosis, and normal kidney function [46]. Hyperkalemia in PHAII is caused by abnormalities in the lysine-deficient protein kinase (WNK) 1 and WNK4, which regulate NCC phosphorylation in the distal nephron. Mutant WNK4 or WNK1 proteins lead to NCC hyperphosphorylation, increasing sodium reabsorption via NCC and reducing sodium delivery to the collecting duct [47]. Consequently, potassium excretion from the principal cells is impaired, resulting in hyperkalemia.

Calcineurin inhibitors (CNIs) are commonly used in nephrology, such as for nephrotic syndrome and as antirejection therapy for transplant recipients. Calcineurin regulates NCC dephosphorylation through a calmodulin-dependent pathway [48] and by inhibiting the WNK-SPAK (Ste20-proline-alanine-rich kinase) pathway [49]. Therefore, CNIs increase the amount of phosphorylated NCC, decrease sodium reabsorption in the collecting ducts, reduce potassium excretion from the principal cells, and can lead to hyperkalemia [50]. Thus, CNIs can propose the same symptoms and electrolyte abnormalities that resemble Gordon syndrome (PHAII). A unique case study introduced a kidney transplant recipient with hypertension and hyperkalemia. Based on the pathophysiology, a thiazide diuretic was found to be effective in this case, leading the authors to conclude that it was “tacrolimus-induced Gordon-like syndrome” [51].

##### Abnormal potassium release from cells

Increased tissue destruction, with causes such as rhabdomyolysis, crush syndrome, and tumor lysis, releases intracellular potassium into the ECF.

### The main cause of hyperkalemia: kidney dysfunction

Typically, in patients with CKD, plasma potassium levels remain below 5.5 mEq/L until the GFR drops below 15 mL/min [52]. A decrease in eGFR by 15 mL/min is associated with an approximately two-fold increase in the likelihood of hyperkalemia [7]. A cohort study investigating serum potassium trajectories in patients transitioning to dialysis found that serum potassium levels did not continuously increase, even with an average GFR of 25 mL/min/1.73 m^2^ and a starting serum potassium level of 4.5 mEq/L. Despite a high incidence of sporadic hyperkalemia (41.1% for serum potassium > 5.5 mEq/L), the crude potassium slope was only + 0.008 mEq/L per year, and after adjusting for background factors, comorbidities, medications, and eGFR, the adjusted potassium slope was negative at −0.15 mEq/L per year [53]. However, this threshold may be exceeded in cases of oliguria, high dietary potassium intake, tissue breakdown, or impaired aldosterone secretion or sensitivity [52]. A cross-sectional study found that acute kidney injury (AKI), potassium-sparing diuretics, CKD stage, and ACEi were significant risk factors for hyperkalemia (> 5.0 mEq/L), with AKI having the highest odds ratio [54]. These studies suggest that even in advanced CKD, the frequency of marked hyperkalemia remains low with appropriate potassium control and monitoring (e.g., potassium restriction and the use of potassium binders), but attention must be paid to AKI-associated complications.

## Tips and pearls of the pathophysiology and causes of hyperkalemia

### Low body mass index (BMI) and frailty as under-recognized risk factors for hyperkalemia

Recent evidence suggests that a low BMI and reduced muscle mass are important but often overlooked risk factors for hyperkalemia. An international cohort study demonstrated an inverse correlation between BMI and serum potassium concentrations, implying that the loss of muscle mass may predispose patients to hyperkalemia [7]. Consistent with this, hyperkalemia has been reported to be more prevalent in patients with lower BMIs [7]. Several mechanisms may explain this association. First, individuals with lower BMIs may have a smaller volume of distribution for drugs, potentially leading to higher serum drug concentrations and an increased risk of hyperkalemia-related toxicity. Second, GFR estimation methods can be influenced by body composition; for instance, the iothalamate method may overestimate this parameter in individuals with a BMI of ≤ 25 kg/m^2^, resulting in an underestimation of the true risk of hyperkalemia [55]. Additionally, frailty and sarcopenia, which are common in the elderly and people with low BMIs, are associated with decreased skeletal muscle mass and, consequently, reduced intracellular potassium storage capacity [56]. This physiological change limits the buffering ability for potassium influx and increases susceptibility to hyperkalemia, particularly when potassium intake is high or potassium excretion is impaired. Furthermore, caution is warranted when administering potassium to patients with a low muscle mass, as serum potassium levels may overshoot due to impaired intracellular uptake. Notably, these associations between low BMI and hyperkalemia have been observed even after stratification by kidney function [7, 55].

### Why does the serum potassium level increase after hospital admission?

As described earlier, potassium excretion is influenced by distal sodium delivery and urine flow within the collecting duct. In clinical practice, patients with CKD may develop hyperkalemia by the second or third day of hospitalization. One predisposing factor is the implementation of a low-salt diet upon admission. This dietary restriction leads to ECF loss within a few days, which reduces the renal blood flow. Additionally, low-salt diets are often also high in potassium (e.g., the DASH [Dietary Approaches to Stop Hypertension] diet). A Japanese cohort study revealed that patients with high salt consumption largely decreased their body weight soon after being admitted and placed on a salt-restriction diet [57]. Consequently, less sodium reaches the distal nephron, impairing urinary potassium excretion and leading to hyperkalemia.

### Dual renin-angiotensin system inhibitor (RASi) should be avoided

Nonsteroidal MRA is effective in inhibiting CKD progression and cardiovascular events in patients with diabetic nephropathy [58, 59]. Based on this evidence, the combination of an ACEi or ARB with a nonsteroidal MRA is recommended. In contrast, there is little or no established evidence supporting the concomitant use of ACEi and ARB [60, 61]. A systematic review and meta-analysis demonstrated that dual RASi (ACEi + ARB) is significantly associated with an increased risk of hyperkalemia and AKI compared to monotherapy [62]. Meanwhile, although the combination of ACEi/ARB and MRA is also associated with hyperkalemia, it does not increase the risk of AKI compared to monotherapy [62]. Additionally, as described in our previous review[63], hyperkalemia is significantly more likely with ACEi/ARB + steroidal MRA than with ACEi/ARB + nonsteroidal MRA [62]. Consequently, the Kidney Disease: Improving Global Outcomes (KDIGO) 2024 Clinical Practice Guideline recommends avoiding any combination of ACEi, ARB, and direct renin inhibitors in patients with CKD, regardless of diabetes status [64].

## Conclusions

Hyperkalemia is commonly defined as a condition in which the serum potassium level is ≥ 5.0 mEq/L. While hyperkalemia evaluation, understanding the dynamics of potassium in the body, including cellular potassium shifts and renal potassium excretion, is important. In addition, distinguishing true hyperkalemia from pseudohyperkalemia is crucial. Abnormal potassium excretion from the kidneys can result from both kidney dysfunction and hypoaldosteronism. Hypoaldosteronism can be further classified into aldosterone deficiency and aldosterone resistance. In summary, hyperkalemia can have causes beyond kidney dysfunction, underscoring the importance of performing a differential diagnosis based on pathophysiology.

## Data Availability

Not applicable.
